# Correlation of Antagonistic Regulation of *leuO* Transcription with the Cellular Levels of BglJ-RcsB and LeuO in *Escherichia coli*

**DOI:** 10.3389/fcimb.2016.00106

**Published:** 2016-09-16

**Authors:** Hannes Breddermann, Karin Schnetz

**Affiliations:** Department of Biology, Institute for Genetics, University of CologneCologne, Germany

**Keywords:** transcription regulator, nucleoid-associated protein, H-NS, H-NS antagonist, feedback regulation

## Abstract

LeuO is a conserved and pleiotropic transcription regulator, antagonist of the nucleoid-associated silencer protein H-NS, and important for pathogenicity and multidrug resistance in *Enterobacteriaceae*. Regulation of transcription of the *leuO* gene is complex. It is silenced by H-NS and its paralog StpA, and it is autoregulated. In addition, in *Escherichia coli leuO* is antagonistically regulated by the heterodimeric transcription regulator BglJ-RcsB and by LeuO. BglJ-RcsB activates *leuO*, while LeuO inhibits activation by BglJ-RcsB. Furthermore, LeuO activates expression of *bglJ*, which is likewise H-NS repressed. Mutual activation of *leuO* and *bglJ* resembles a double-positive feedback network, which theoretically can result in bi-stability and heterogeneity, or be maintained in a stable OFF or ON states by an additional signal. Here we performed quantitative and single-cell expression analyses to address the antagonistic regulation and feedback control of *leuO* transcription by BglJ-RcsB and LeuO using a *leuO* promoter *mVenus* reporter fusion and finely tunable *bglJ* and *leuO* expression plasmids. The data revealed uniform regulation of *leuO* expression in the population that correlates with the relative cellular concentration of BglJ and LeuO. The data are in agreement with a straightforward model of antagonistic regulation of *leuO* expression by the two regulators, LeuO and BglJ-RcsB, by independent mechanisms. Further, the data suggest that at standard laboratory growth conditions feedback regulation of *leuO* is of minor relevance and that silencing of *leuO* and *bglJ* by H-NS (and StpA) keeps these loci in the OFF state.

## Introduction

LeuO is a conserved and pleiotropic LysR-type transcription factor that has been best characterized in *Escherichia coli* and *Salmonella enterica*. LeuO functions both as activator and as repressor, and is presumably a tetramer, similar to other LysR-type regulators (Maddocks and Oyston, 2008; Guadarrama et al., 2014). LeuO is a master regulator with more than 100 target loci, and supposedly an important H-NS antagonist, since many LeuO-activated loci are H-NS repressed (Ueguchi et al., 1998; Chen et al., 2003; Chen and Wu, 2005; De la Cruz et al., 2007; Stoebel et al., 2008; Stratmann et al., 2008, 2012; Shimada et al., 2011; Dillon et al., 2012; Ishihama et al., 2016). In addition, genomics data revealed a significant overlap of co-regulation by LeuO and H-NS both in *E. coli* and in *S. enterica*, where 78 and 40%, respectively, of the LeuO targets are H-NS bound (Shimada et al., 2011; Dillon et al., 2012; Ishihama et al., 2016). H-NS represses transcription by formation of extended complexes on the DNA (Dillon and Dorman, 2010; Landick et al., 2015; Winardhi et al., 2015). For activation of H-NS repressed loci by LeuO several mechanisms have been proposed including alteration of the repressing H-NS nucleoprotein-complex, the prevention of spreading of the H-NS complex, and competition with H-NS for DNA binding (Chen and Wu, 2005; Shimada et al., 2011; Dillon et al., 2012). The biological role of LeuO is pleiotropic. LeuO is relevant for pathogenicity in *S. enterica*, for biofilm formation in *Vibrio cholerae* and *E. coli*, as well as the acid stress response and multidrug efflux in *E. coli* (Stoebel et al., 2008; Shimada et al., 2009, 2011; Dillon et al., 2012). Further, LeuO activates expression of the H-NS repressed genes coding for the CRISPR/Cas immunity system in *E. coli* and *S. enterica* (Pul et al., 2010; Westra et al., 2010; Medina-Aparicio et al., 2011).

**Figure 1:**
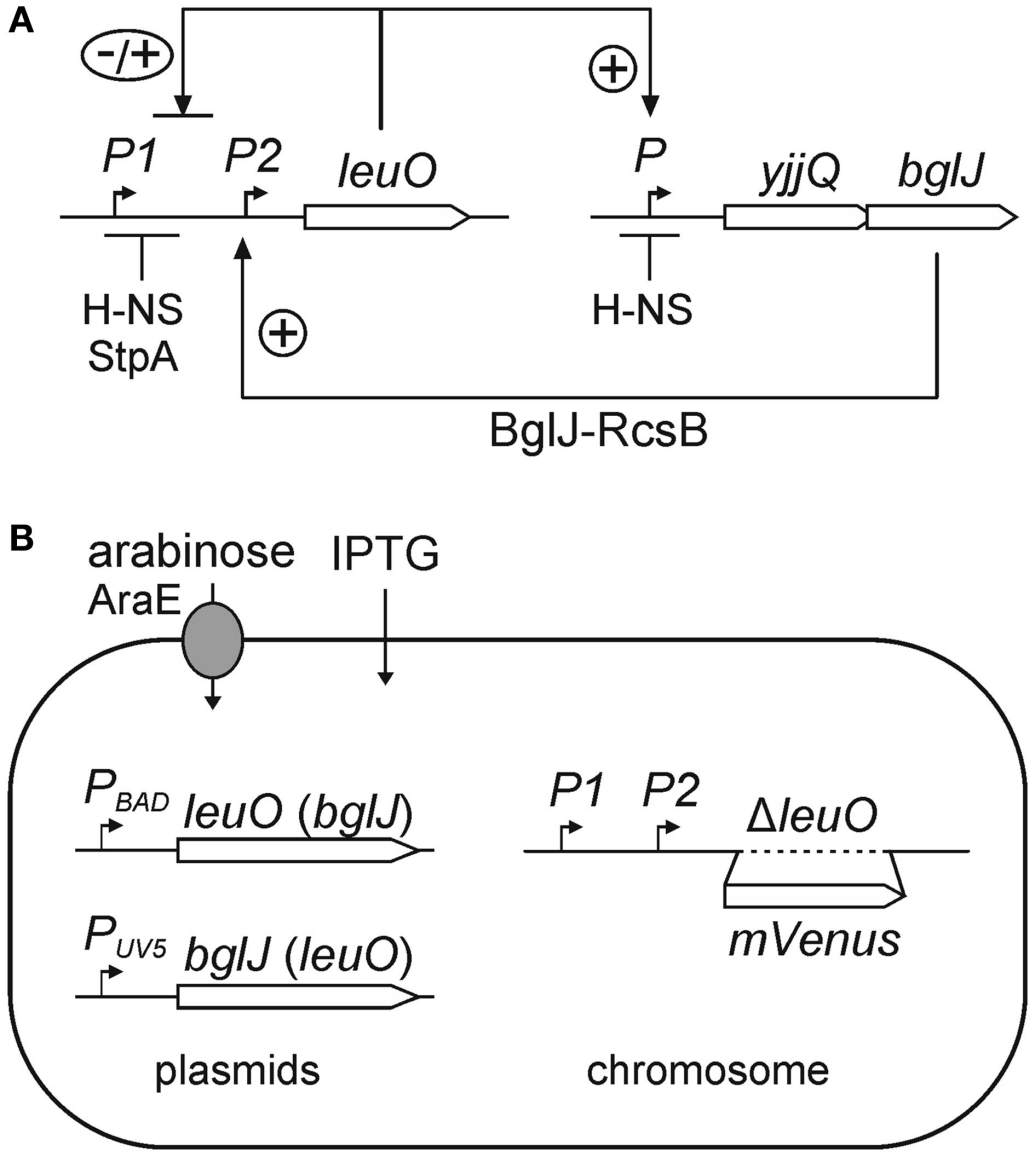
**(A)** Regulation of *leuO* by interlocked double-positive and negative feedback loops. Transcription of *leuO* is repressed by H-NS and StpA, and is activated by the BglJ-RcsB heterodimer. LeuO activates transcription of the *yjjQ-bglJ* operon that is also repressed by H-NS. Mutual positive regulation represents a double-positive feedback loop. In addition, LeuO inhibits activation of the *leuO* promoter *P2* by BglJ-RcsB resembling a negative feedback. **(B)** Experimental system for analyzing regulation of *leuO* transcription by BglJ-RcsB and LeuO. To monitor *leuO* transcription a *PleuO mVenus* fusion was constructed by replacement of the native *leuO* gene with *mVenus*. The chromosomal copy of *bglJ* was deleted (allele Δ[*yjjP-yjjQ-bglJ*]) to avoid feedback regulation via LeuO. BglJ and LeuO were provided by two sets of compatible plasmids that are pKES303 (*P*_*BAD*_ *leuO*, p15A-*ori*) and pKETS26 (*P*_*UV*5_ *bglJ*, pSC-*ori*) or plasmid pKES302 (*P*_*BAD*_ *bglJ*) and pKETS25 (*P*_*UV*5_ *leuO*). Expression of *bglJ* and *leuO*, respectively, was induced with gradually increasing concentrations of the inducers arabinose and IPTG, respectively. To avoid feedback regulation by arabinose the strain background is Δ(*araC araBAD*) Δ*araH-F*, *P*_*cp*8_ *araE* resulting in constitutive expression of the arabinose transporter AraE. In addition, the *lac* genes were deleted, allele Δ(*lacI-lacZYA*), for enabling gradual induction by IPTG.

In accordance with the pleiotropic role of LeuO, transcription of *leuO* is tightly controlled. Under laboratory conditions the *leuO* gene is repressed by H-NS and by the H-NS paralog StpA, and thus the *leuO* gene is silent in *E. coli* and *S. enterica* (Klauck et al., 1997; Chen et al., 2001). Moderate upregulation of *leuO* expression was observed in stationary phase and under amino acid starvation (Fang and Wu, 1998; Fang et al., 2000; Majumder et al., 2001; Shimada et al., 2011; Dillon et al., 2012). In addition, positive autoregulation by LeuO and transcriptional coupling of *leuO* expression to expression of neighboring genes by DNA supercoiling has been reported (Fang and Wu, 1998; Chen et al., 2003). Furthermore, in *E. coli leuO* is activated by the heterodimeric transcription regulator BglJ-RcsB (Stratmann et al., 2012). Activation of *leuO* by BglJ-RcsB is inhibited by LeuO, and LeuO represses *leuO* transcription in *hns* and in *hns stpA* mutants (Figure 1A). Thus, LeuO is also a negative autoregulator (Stratmann et al., 2012). The *leuO* gene is preceded by at least two promoters (*P1* and *P2*) which are repressed by H-NS and StpA and negatively autoregulated by LeuO in *hns stpA* mutants; the *P2* promoter is activated by BglJ-RcsB (Stratmann et al., 2012). BglJ-RcsB is a heterodimer that activates transcription of various loci in *E. coli* (Venkatesh et al., 2010; Stratmann et al., 2012; Salscheider et al., 2014). BglJ-RcsB consists of RcsB, the response regulator of the Rcs two-component phosphorelay system (Majdalani and Gottesman, 2005), and BglJ, which has initially been found as an activator of the *bgl* operon (Giel et al., 1996). Further, BglJ-RcsB is active independent of phosphorylation of RcsB by the Rcs phosphorelay (Venkatesh et al., 2010; Stratmann et al., 2012; Pannen et al., 2016).

Intriguingly, activation of *leuO* by BglJ-RcsB is one element of a presumptive double-positive feedback loop, since LeuO in turn activates expression of the *yjjQ-bglJ* operon that is likewise H-NS repressed (Stratmann et al., 2008). This double-positive feedback loop is interlocked with a negative feedback loop which is based on negative autoregulation by LeuO (Figure 1). Such a network motif can function like a switch that is stable both in the OFF as well as in the ON state. Often an external signal locks such feedback loops in one state. Further, bi-stability resulting in population heterogeneity and oscillation can be based on interlocked positive and negative feedback loops (Angeli et al., 2004; Alon, 2007; Shoval and Alon, 2010).

In this study we addressed the antagonistic regulation of *leuO* transcription by BglJ-RcsB and LeuO, which is presumably a crucial element in the complex control of *leuO* expression. For quantitative and single-cell expression analysis, we established a reporter fusion of the *leuO* promoter region (*P*_*leuO*_) to *mVenus* and expressed *bglJ* and *leuO in trans* using tightly controlled and gradually inducible plasmidic expression systems. Expression analyses of the *P*_*leuO*_ *mVenus* reporter at steady state growth conditions revealed uniform expression. The level of *leuO* expression correlates with the relative cellular concentration of BglJ and LeuO. The data are in agreement with a straightforward model of antagonistic regulation by the two regulators that act independently of each other.

## Results

### Experimental system for analyzing regulation of *leuO* expression by BglJ and LeuO

The regulation of *leuO* transcription by BglJ-RcsB and LeuO is an important element in the control of the LeuO master regulator. To address regulation of *leuO* transcription that is directed by at least two promoters (*P*_*leuO*_) in dependence of the concentrations of BglJ and LeuO, a suitable experimental system was established. First, the *mVenus* reporter gene (coding for the yellow fluorescent protein mVenus) was fused to the *leuO* promoter-regulatory region by replacement of the *leuO* gene resulting in allele *P*_*leuO*_ *mVenus*, Δ*leuO* (Figure 1B). Second, BglJ and LeuO were ectopically expressed from two different sets of plasmids. In one plasmid set, *bglJ* was expressed under control of the IPTG-inducible *lacUV5* promoter (*P*_*UV*5_) using low-copy plasmid pKETS26 (pSC origin of replication), and *leuO* was expressed under control of the arabinose-inducible *P*_*BAD*_ promoter using the low to medium copy plasmid pKES303 (pBAD30-derived, p15A origin of replication). In the other plasmid set, *bglJ* was expressed under control of the *P*_*BAD*_ promoter (pKES302, p15A-*ori*) and *leuO* under control of IPTG-inducible *P*_*tac*_ promoter (pKEHB27, pSC-*ori*). The genes encoding the AraC and the LacI regulators, respectively, are also carried on these plasmids. Additionally, the *yjjQ-bglJ* operon was deleted resulting in allele Δ(*yjjP-yjjQ-bglJ*) to ensure that only plasmid-encoded BglJ is present in the cell. Note that RcsB is not limiting for activation of *leuO* and other loci by BglJ-RcsB (Salscheider et al., 2014; Pannen et al., 2016). Third, to allow controlled and finely tunable expression of *bglJ* and *leuO* directed by the arabinose-inducible P_*BAD*_ promoter and the IPTG-inducible *P*_*UV*5_ and *P*_*tac*_ promoters, respectively, additional mutations and modifications were introduced into the reporter strain (Figure 1B). The *P*_*UV*5_ promoter is gradually induced over a range of inducer concentrations (IPTG) when the lactose permease gene *lacY* is deleted (Jensen et al., 1993). Therefore, the *lacZYA* operon and the *lacI* gene were deleted in the reporter strain resulting in allele Δ(*lacI-lacZYA*) (Table 1). Likewise, the arabinose regulon was modified to ensure a gradual induction of the *P*_*BAD*_ promoter with arabinose, as described before (Khlebnikov et al., 2001; Kogenaru and Tans, 2014). Briefly, the *P*_*BAD*_ promoter is known to have a stochastic behavior when induced with arabinose. This stochastic behavior is caused by the *araE* and *araFGH* genes encoding the arabinose transporters, because induction of the transporter genes by arabinose leads to a higher arabinose uptake and thus positive feedback (Siegele and Hu, 1997; Megerle et al., 2008). In addition, a negative feedback caused by fermentation of intracellular arabinose through the AraBAD enzymes leads to a non-gradual induction (Siegele and Hu, 1997). To avoid the negative and positive feedback, the *araC* gene and the *araBAD* and *araFGH* operons were deleted. Further, the low affinity arabinose transporter *araE* was put under the control of constitutive promoter *P*_*cp*8_, as described (Khlebnikov et al., 2001; Kogenaru and Tans, 2014). The genotype of the resulting reporter strain U69 is *P*_*leuO*_ *mVenus* Δ*leuO* Δ(*yjjP-yjjQ-bglJ*) φ(Δ*araE*p *P*_*cp*8_ *araE*) Δ(*araH-F*) Δ(*araC-araBAD*) Δ(*lacI-lacZYA*) (Table 1). Using this strain the expression level of *P*_*leuO*_ *mVenus* was measured by flow-cytometry to quantify the cellular fluorescence in the population. Further, to ensure steady state conditions, cultures were grown in nutrient-poor tryptone medium. In this medium cultures that were inoculated from fresh overnight cultures to OD_600_ of 0.05 reached an OD_600_ of about 0.7–1 after 5 h of growth.

**Table 1:** ***E. coli* K12 strains**.

**Strain**	**Genotype**	**Reference/Construction**
BW27269	BW25113 Δ(araH-araF)572_kan_ = CGSC strain #7877 (laboratory storage number T1857)	Khlebnikov et al., 2001
BW27270	BW25113 ΔaraEp-531_kan_ φP_cp8_araE535 (= _kan_P_cp8_araE) = CGSC strain #12117 (laboratory storage number T1858)	Khlebnikov et al., 2001
S3974	BW30270 ilvG^+^ [ = MG1655 rph^+^ ilvG^+^] (non-motile)	Venkatesh et al., 2010
S4197	BW30270 ilvG^+^ ΔlacZ [ = MG1655 rph^+^ ilvG^+^ ΔlacZ] (non-motile)	Venkatesh et al., 2010
T17	S4197 Δ(yjjP-yjjQ-bglJ)_cm_	parent of strain T23 in (Stratmann et al., 2012)
T1024	S3974 Δ(lacI-lacZYA)_FRT_	S3974 × PCR S911/S937 (pKD3); × pCP20
T1037	T1024 P_leuO_− leuO::mVenus_cm_	T1024 × PCR T547/T548 (pKES292)
T1094	S3974 P_leuO_mVenus_cm_, ΔleuO	S3974 × PCR T585/T548 (pKES292)
T1095	S3974 P_leuO_mVenus_kan_, ΔleuO	S3974 × PCR T585/T548 (pKES293)
T1241	BW30270 ilvG^+^ (motile)	Pannen et al., 2016
T1902	T1241 P_molR_mVenus_cm_	T1241 × PCR T946/T947 (pKES292)
U1	T1241 Δ(araC-araBAD)	T1241 × pKETS27
U3	T1241 Δ(araC-araBAD) Δ(lacI-lacZYA)	U1 × pKETS28
U9	U3 P_leuO_mVenus_kan_, ΔleuO	U3 × T4*GT7* (T1095)
U11	U3 Δ(yjjP-yjjQ-bglJ)_cm_	U3 × T4*GT7* (T17)
U15	U3 Δ(yjjP-yjjQ-bglJ)_FRT_	U11 × pCP20
U16	U3 P_leuO_mVenus_kan_, ΔleuO Δ(yjjP-yjjQ-bglJ)_cm_	U9 × T4*GT7* (T17)
U20	U3 P_leuO_mVenus_FRT_, ΔleuO Δ(yjjP-yjjQ-bglJ)_FRT_	U16 × pCP20
U47	U3 _kan_P_cp8_-araE	U3 × T4*GT7* (BW27270)
U49	U3 Δ(yjjP-yjjQ-bglJ)_FRT_ _kan_P_cp8_araE	U15 × T4*GT7* (BW27270)
U51	U3 P_leuO_mVenus_FRT_, ΔleuO Δ(yjjP-yjjQ-bglJ)_FRT_ _kan_P_cp8_araE	U20 × T4*GT7* (BW27270)
U53	U3 P_cp8_araE	U47 × pCP20
U55	U3 Δ(yjjP-yjjQ-bglJ)_FRT_ P_cp8_araE	U49 × pCP20
U57	U3 P_leuO_mVenus_FRT_, ΔleuO Δ(yjjP-yjjQ-bglJ)_FRT_ P_cp8_araE	U51 × pCP20
U59	U3 P_cp8_araE Δ(araH-araF)_kan_	U53 × T4*GT7* (BW27269)
U61	U3 Δ(yjjP-yjjQ-bglJ)_FRT_ P_cp8_araE Δ(araH-araF)_kan_	U55 × T4*GT7* (BW27269)
U62	U3 Δ(yjjP-yjjQ-bglJ)_FRT_ P_cp8_araE Δ(araH-araF)_kan_	U56 × T4*GT7* (BW27269)
U63	U3 P_leuO_mVenus_FRT_, ΔleuO Δ(yjjP-yjjQ-bglJ)_FRT_ P_cp8_araE Δ(araH-araF)_kan_	U57 × T4*GT7* (BW27269)
U65	U3 P_cp8_araE Δ(araH-araF)_FRT_	U59 × pCP20
U67	U3 Δ(yjjP-yjjQ-bglJ)_FRT_ P_cp8_araE Δ(araH-araF)_FRT_	U61 × pCP20
U69	U3 P*_leuO_*mVenus_FRT_, ΔleuO Δ(yjjP-yjjQ-bglJ)_FRT_ P_cp8_araE Δ(araH-araF)_FRT_	U63 × pCP20
U76	U65 P_molR_mVenus_FRT_	U65 × T4*GT7* (T1092); x pCP20
U92	U3 P_cp8_araE Δ(araH-araF)_FRT_ P*_leuO_*leuO::mVenus_cm_	U65 × T4*GT7* (T1037)
U93	U3 P_cp8_araE Δ(araH-araF)_FRT_ P*_leuO_*mVenus_cm_, ΔleuO	U65 × T4*GT7* (T1094)
U94	U3 P*cp*8araE Δ(araH-araF)_FRT_ P*_leuO_*leuO::mVenus_FRT_	U92 × pCP20
U95	U3 P*cp*8araE Δ(araH-araF)_FRT_ P*_leuO_*mVenus_FRT_, ΔleuO	U93 × pCP20
U96	U3 Δ(yjjP-yjjQ-bglJ)_FRT_ P_cp8_araE Δ(araH-araF)_FRT_ P*_leuO_*leuO::mVenus_cm_	U67 × T4*GT7* (T1037)
U97	U3 Δ(yjjP-yjjQ-bglJ)_FRT_ P_cp8_araE Δ(araH-araF)_FRT_ P*_leuO_*leuO::mVenus_FRT_	U96 × pCP20

Alleles Δ(araC-araBAD) and Δ(lacI-lacZYA) were constructed by homologous recombination, as described (Hamilton et al., 1989), using rep_ts_ plasmids pKETS27 and pKETS28, respectively. Transcriptional fusions of mVenus to the leuO promoter (P_leuO_-mVenus) and downstream of the leuO gene (P_leuO_-leuO::mVenus) were constructed by Red-Gam mediated recombination, as described (Datsenko and Wanner, 2000). Red-Gam expression carried on plasmid pKD46 was induced with 10 mM arabinose. Plasmids pKES292 and pKES293 were used as templates for amplification of mVenus-FRT-kan/cm-FRT fragments. The oligonucleotides used for generating the PCR fragments are indicated by “PCR T547/T548.” Deletion of the lac genes in strain T1024 was constructed as described (Datsenko and Wanner, 2000) using oligonucleotides S911/S937 for generating the PCR fragment of pKD3 as template. Resistance cassettes flanked by FRT (Flp-recombinase target) sites were deleted using temperature sensitive plasmid pCP20, as described (Datsenko and Wanner, 2000). The transfer of alleles by transduction using phage T4GT7 is indicated by “x T4GT7 (donor strain).” All alleles were confirmed by PCR. Alleles P_leuO_-leuO::mVenus_cm_ in strain T1037, P_leuO_mVenus_cm_ in strain T1094 and P_leuO_mVenus_kan_ in strain T1095 were confirmed by sequencing. Further designations are cm = chloramphenicol resistance, kan = kanamycin resistance, FRT = Flp recombinase target site, rep_ts_ = temperature sensitive replication.

### Regulation of *leuO* promoter by BglJ–RcsB and by LeuO

**Figure 2:**
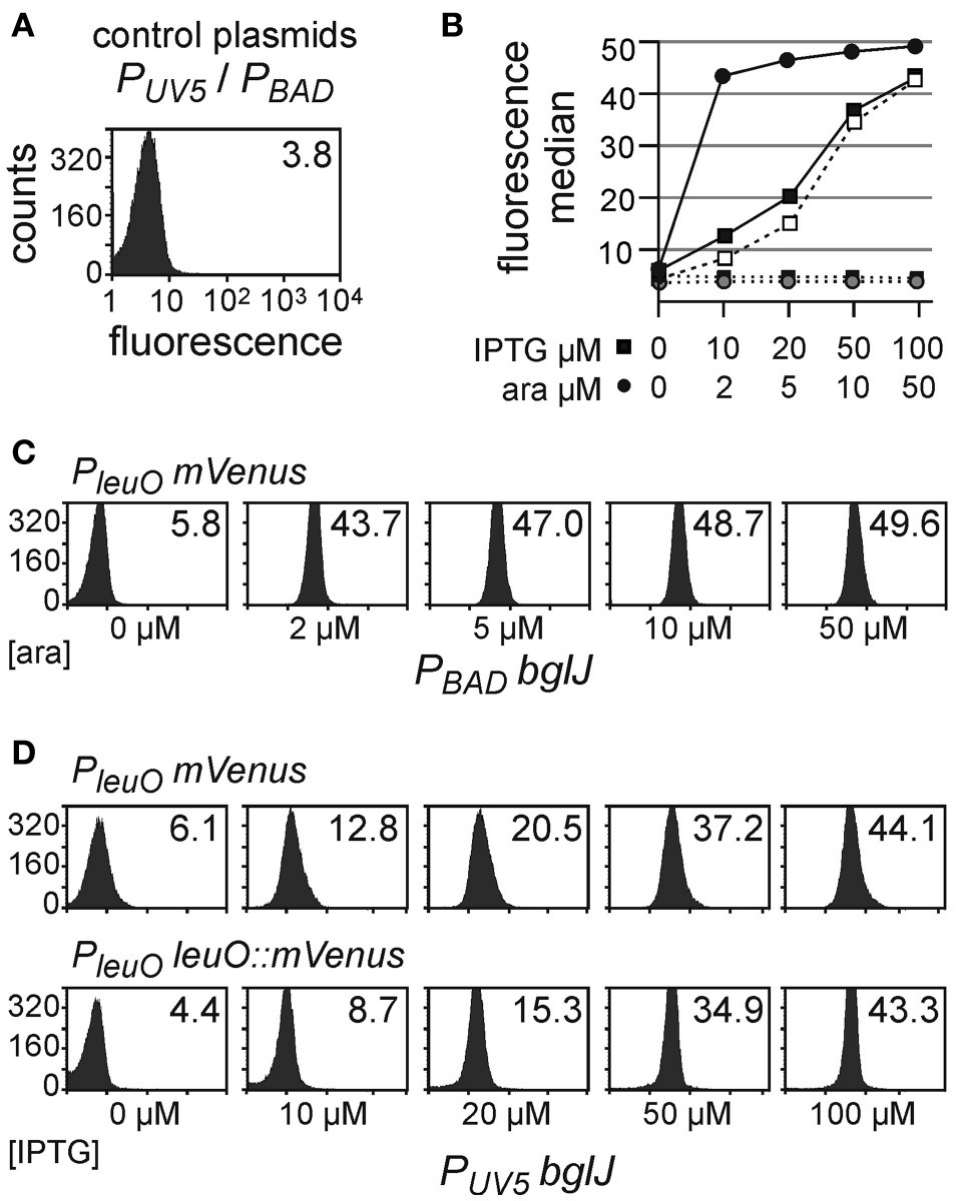
**Activation of *leuO* transcription by BglJ**. Expression of *P*_*leuO*_ *mVenus* (in strain U69) and *P*_*leuO*_ *leuO::mVenus* (strain U97) transcriptional fusions was determined by flow cytometry in absence and presence of the transcriptional activator BglJ, which was provided by plasmids. Expression was analyzed after 5 h of growth in tryptone medium without and with indicated inducer concentrations at an optical density OD_600_ of approximately 0.7–1. **(A)** Fluorescence intensity directed by *P*_*leuO*_ *mVenus* in individual cells of transformants of strain U69 with the empty vectors pKETS24 (*P_UV5_* in pSC-*ori*) and pBAD30 (*P_BAD_* in p15A-*ori*). Yellow fluorescence (X-axis) is given in arbitrary units and the Y-axis gives the number of cells that were counted. The median of the fluorescence intensity is given in the upper right corner of the graph. **(B)** Plot of the median fluorescence values that are shown in **(C)** (solid line with filled dots) and **(D)** (solid line with filled squares *P*_*leuO*_ *mVenus* and dashed line with open squares *P*_*leuO*_ *leuO::mVenus*). In addition, median fluorescence values of transformants of vector controls are shown (pKETS24, *P*_*UV5*_ as dotted line and filled squares, and pBAD30, *P*_*BAD*_ dotted line with gray dots). **(C)** Fluorescence intensity of transformants of strain U69 with plasmids pKES302 (*P*_*BAD*_ *bglJ* in p15A-*ori*) and pKETS24 (*P*_*UV5*_ in pSC-*ori*). The arabinose concentration used for induction of *bglJ* expression is given underneath the panels. **(D)** Fluorescence intensity of transformants of strain U69 (*P*_*leuO*_ *mVenus*) with plasmids pKETS26 (*P*_*UV5*_ *bglJ* in pSC-*ori*) and pBAD30 (*P*_*BAD*_ in p15A-*ori*), as well as of strain U97 (*P*_*leuO*_ *leuO::mVenus*). The IPTG concentration used for induction of *bglJ* expression is given underneath the panels. Shown are representative data.

First, activation of the *P*_*leuO*_ *mVenus* fusion by BglJ-RcsB was tested. To this end, the reporter strain U69 was transformed with low-copy plasmid pKETS26 carrying *bglJ* under control of the IPTG-inducible *P*_*UV*5_ promoter (*P*_*UV*5_ *bglJ*, pSC-*ori*), and with plasmid pKES302 carrying *bglJ* under control of the arabinose-inducible *P*_*BAD*_ promoter (*P*_*BAD*_ *bglJ*, p15A-*ori*), respectively (Figure 2). Expression of *bglJ* was either not induced or induced by gradually increasing inducer concentrations. The analysis of *P*_*leuO*_ *mVenus* expression by flow-cytometry revealed that gradual induction of *P*_*BAD*_ *bglJ* expression (plasmid pKES302) with 2 μM–50 μM arabinose resulted in full activation of *P*_*leuO*_ *mVenus* even at the very low arabinose concentration of 2 μM (Figures 2B,C). Induction of *P*_*BAD*_ *bglJ* with 100 μM arabinose or higher concentrations caused growth defects. However, induction of *P*_*UV*5_ *bglJ* with IPTG concentration ranging from 10 μM to 100 μM led to a gradual increase in expression of *P*_*leuO*_ *mVenus* and this increase was uniform in the population (Figures 2B,D). The presence of the *P*_*UV*5_ *bglJ* or the *P*_*BAD*_ *bglJ* plasmids *per se* did not cause a significant increase in expression of *P*_*leuO*_ *mVenus* (Figures 2B–D). Likewise, IPTG or arabinose induction of transformants of the empty vectors pBAD30 and pKETS24, respectively, had no effect (Figure 2B). Taken together these data confirm activation of *leuO* transcription by BglJ-RcsB, they suggest that low cellular levels of BglJ are sufficient for activation, and that the *P*_*UV*5_ *bglJ* plasmid is suitable for gradual induction of *bglJ*, while the *P*_*BAD*_ *bglJ* plasmid is not suitable.

**Figure 3:**
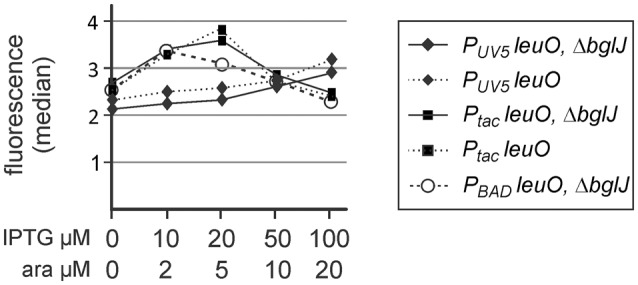
**Autoregulation of *leuO* transcription**. Fluorescence expression levels directed by the *P*_*leuO*_ *mVenus* fusion were determined by flow cytometry. The *P*_*leuO*_ *mVenus* reporter strains U69 carrying a deletion of *bglJ* (Δ*yjjP-yjjQ-bglJ*) and its isogenic wild-type *bglJ*^+^ derivative U95 were transformed with plasmids pKETS25 (pSC-*ori*) that carries *leuO* under control of *P*_*UV*5_, pKEHB27 (p15A-*ori*) that carries *leuO* under control of *P*_*tac*_, and pKES303 (p15A-*ori*) that carries *leuO* under control of *P*_*BAD*_. The fluorescence median is plotted against the inducer concentration. Expression was analyzed by flow cytometry after 5 h of growth in trypton medium, IPTG, and arabinose were added at the indicated concentrations. Statistical analysis suggests that the difference in the expression level is not significant (*P* > 0.05).

Second, autoregulation of *P*_*leuO*_ *mVenus* by LeuO was analyzed using the *leuO* providing plasmids *P*_UV5_ *leuO* (pKETS25, pSC-*ori*) and *P*_tac_ *leuO* (pKEHB27, pSC-*ori*) which carry *leuO* under control of the IPTG-inducible *P*_UV5_ and *P*_*tac*_ promoters, respectively. In addition, a *P*_*BAD*_ *leuO* plasmid (pKES303, p15A-*ori*) was used. The promoter *P*_*UV*5_ (carrying the *UV5* mutation in the—10 box and the *lacL8* mutation in the CRP-binding site) is ~10 times weaker than the *P*_tac_ promoter (Lanzer and Bujard, 1988), while the tightly regulated *P*_*BAD*_ *leuO* plasmid presumably directs similar levels of LeuO as the *P*_tac_ *leuO* plasmid considering that the *P*_BAD_ promoter is approximately 3 fold weaker than *P*_tac_ and that the copy number of the *P*_BAD_ plasmid (pKES303, p15A-*ori*) is ~3-fold higher than the copy number of the pSC-derived *P*_tac_ plasmid (Guzman et al., 1995). Flow cytometry revealed a slight increase in *P*_*leuO*_ *mVenus* expression at low levels of induction of plasmidic *leuO* (Figure 3). The data seem in agreement with weak positive autoregulation that was reported previously (Fang and Wu, 1998; Chen et al., 2003), but are statistically not significant (student's *t*-test, *P*-value > 0.05).

### Antagonistic regulation of the *leuO* promoter by BglJ–RcsB and by LeuO

**Figure 4:**
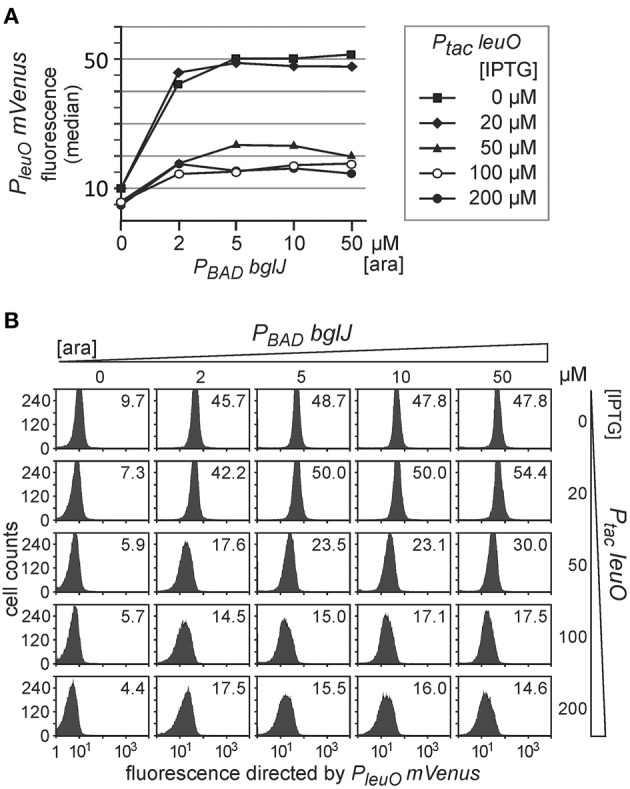
**Antagonistic regulation of *P*_*leuO*_*mVenus* transcription by BglJ-RcsB and LeuO**. Fluorescence of transformants of *P*_*leuO*_ *mVenus* strain U69 with plasmids pKES302 (*P*_*BAD*_ *bglJ*) and pKEHB27 (*P*_*tac*_ *leuO*) was monitored by flow cytometry. **(A)** The median fluorescence is plotted against the arabinose concentration used for induction of *bglJ*. Each line graph represents the set of data obtained of cultures grown with the specified IPTG concentration used for induction of *leuO*. **(B)** Flow cytometry data of cultures grown with increasing arabinose (rows) and IPTG (columns) concentration. Plotted in each panel are the cell counts against the fluorescence intensity. The fluorescence distribution in each panel is in agreement with uniform expression within the population. The fluorescence median that is plotted in **(A)** is given within each panel. Cultures were inoculated from overnight cultures to an OD_600_ of 0.05 and grown for 5 h in 10 ml tryptone medium containing ampicillin, chloramphenicol, as well as IPTG and arabinose at the indicated concentrations.

Next we addressed antagonistic regulation of *P*_*leuO*_ *mVenus* by BglJ-RcsB and by LeuO. To this end, the *P*_*leuO*_ *mVenus* reporter strain U69 was transformed with the two sets of *leuO* and *bglJ* expressing plasmids. First we analyzed antagonistic regulation of *leuO* transcription using the plasmid set, in which *bglJ* is expressed under control of the *P*_*BAD*_ promoter (*P*_*BAD*_ *bglJ*, pKES302) and *leuO* is expressed under control of the *P*_*tac*_ promoter (*P*_*tac*_ *leuO*, pKEHB27). Induction of *bglJ* expression with 2 μM–50 μM arabinose caused full activation of *P*_*leuO*_ *mVenus* (Figure 4), irrespective of the arabinose concentration, as shown above (Figure 2). Simultaneous induction of *leuO* by IPTG strongly reduced BglJ-RcsB-mediated activation of *P*_*leuO*_ *mVenus*, but even full induction of plasmidic *leuO* expression with 200 μM IPTG did not completely abrogate BglJ-RcsB-mediated activation (Figure 4). These results indicate that the level of BglJ provided by the *P*_*BAD*_ *bglJ* plasmid is above a threshold up to which LeuO can fully inhibit BglJ-RcsB activation. Since the *P*_*BAD*_ *bglJ* plasmid does not allow gradual activation, this plasmid set does not seem suitable for gradual induction of both regulators.

**Figure 5:**
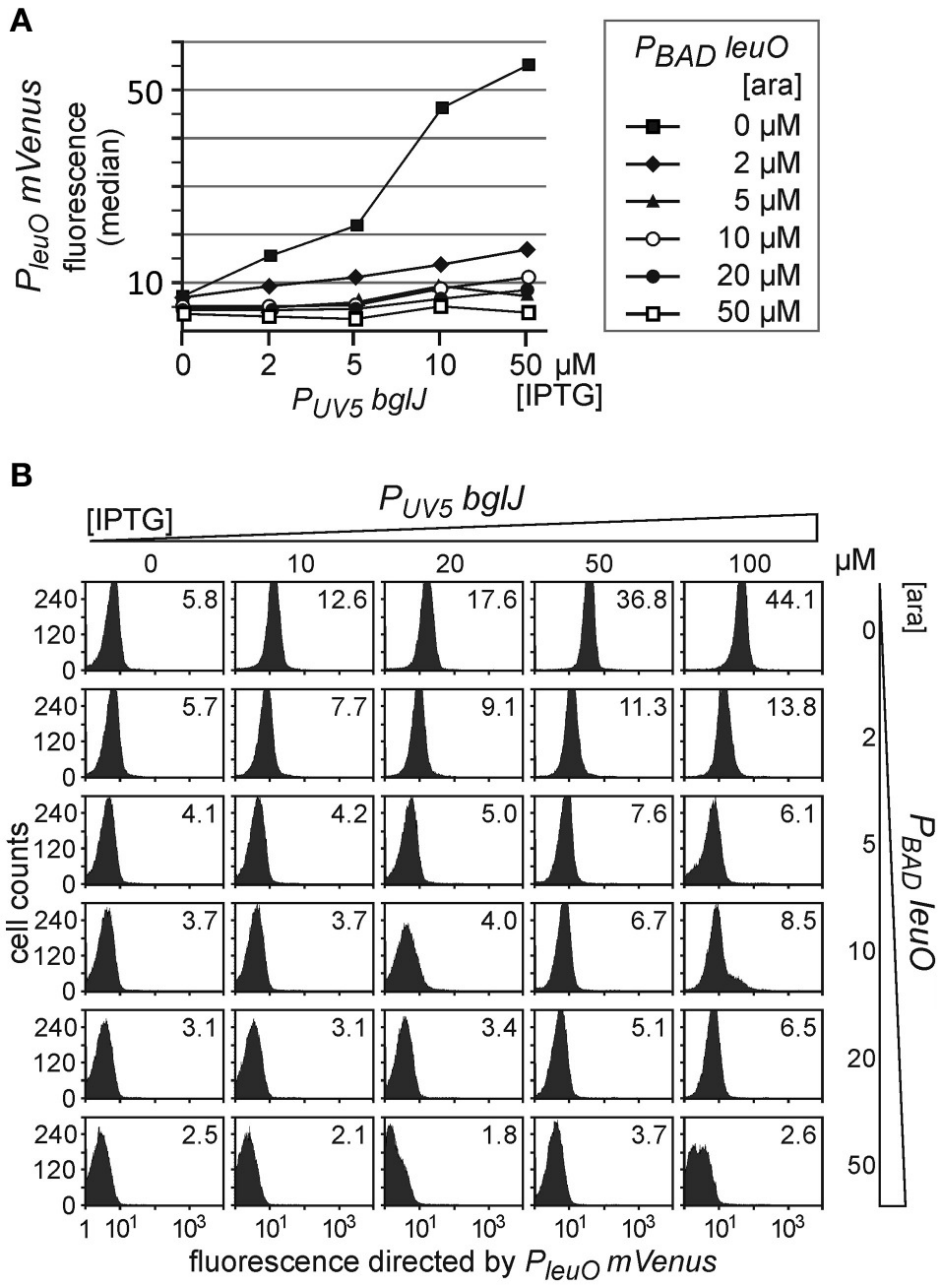
**Antagonistic regulation of *P*_*leuO*_*mVenus* transcription by BglJ and LeuO**. Transformants of *P*_*leuO*_ *mVenus* strain U69 with plasmids pKES303 (*P*_*UV*5_ *bglJ*) and pKEHB28 (*P*_*BAD*_ *leuO*) were grown for 5 h in tryptone medium containing arabinose and IPTG at the indicated concentrations. Fluorescence was monitored by flow cytometry. **(A)** The median fluorescence is plotted against the IPTG concentration that was used for induction of *bglJ*. Each line graph represents the set of median fluorescence data that was obtained when plasmidic *leuO* was induced with the indicated arabinose concentrations. **(B)** Flow cytometry data of cultures grown with increasing IPTG (rows) and arabinose (columns) concentration (presentation of data as in Figure 4).

Second, we analyzed antagonistic regulation of *P*_*leuO*_ *mVenus* using the reverse set of plasmids that includes *P*_*UV*5_ *bglJ* (pKETS26) and *P*_*BAD*_ *leuO* (pKES303) (Figure 5). With this set of plasmids expression levels of BglJ are lower and gradual induction of *bglJ* by IPTG resulted in a gradual increase in activation of the *P*_*leuO*_ *mVenus* fusion by BglJ-RcsB (Figure 5, compare with data in Figure 2). Simultaneous gradual induction of plasmidic *P*_*BAD*_ *leuO* with arabinose and of *P*_*UV*5_ *bglJ* with IPTG led to a uniform decrease of expression of *P*_*leuO*_ *mVenus* in the whole population as compared to level of activation by BglJ-RcsB alone (Figure 5). Induction of *leuO* with an arabinose concentration of 50 μM was sufficient to completely abrogate activation by BglJ-RcsB (bottom right panel, Figure 5B). A plot of the median values of the flow cytometry results visualizes the gradual effects (Figure 5A).

**Figure 6:**
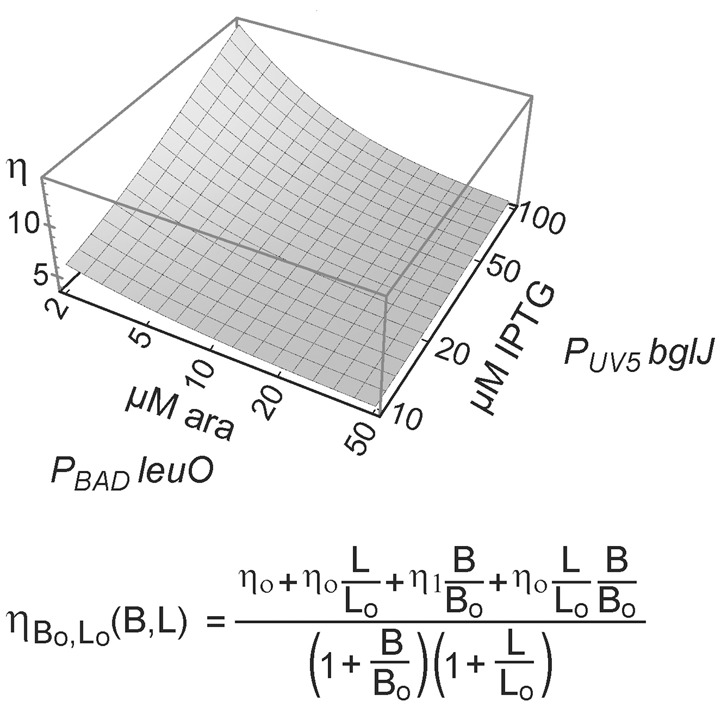
**Modeling of antagonistic regulation of *leuO* transcription by BglJ-RcsB and LeuO**. To describe the transcription rate directed by *P*_*leuO*_ in dependence of the concentration of BglJ and LeuO, a thermodynamic model based on Michaelis-Menten kinetics was used. In this model it was assumed that BglJ and LeuO bind and regulate *leuO* transcription independently of each other. Median fluorescence values of flow cytometry data (Figure 5) were fitted to the function (bottom) describing *leuO* promoter activity in dependence of promoter occupancy by BglJ and LeuO. Fitting of the parameters to the experimental data by nonlinear regression according to (Fox and Weisberg, 2011) yielded *P*-values < 0.001. The data were plotted with Mathematica (Wolfram Research) using logarithmic scales for induction of plasmidic *leuO* with arabinose (ara) and of plasmidic *bglJ* with IPTG.

Taken together, the data confirm that LeuO counteracts activation of the *leuO* promoter by BglJ-RcsB. Further, the data show that antagonistic regulation of the *leuO* promoters by LeuO and by BglJ-RcsB depends on the relative concentration of BglJ and LeuO, and the data indicate that BglJ-RcsB-mediated activation of *P*_*leuO*_ *mVenus* is inhibited by LeuO only if BglJ levels are rather low. The experimental data shown in Figure 5 were used to describe *P*_*leuO*_ activity in dependence of the concentration of BglJ and LeuO by a thermodynamic model based on Michaelis-Menten kinetics. In this model it was assumed that BglJ and LeuO regulate *P*_*leuO*_ independently of each other. Fitting of the function to the experimental data was significant (*P*-value < 0.001) (function plotted in Figure 6).

### Analysis of feedback regulation of *leuO* via *yjjQ–bglj* and by LeuO

Next we addressed the relevance of the presumptive double-positive feedback regulation of *leuO* and *bglJ* by including the native gene of one of these two players, while providing the other one by the expression plasmid. In particular, we analyzed whether presence of the native *yjjQ-bglJ* operon that is activated by LeuO results in enhanced *P*_*leuO*_ *mVenus* expression, when LeuO is provided *in trans*. Second, we tested whether the presence of native *leuO* might affect activation of *P*_*leuO*_ by BglJ-RcsB.

For determining whether activation of the H-NS repressed *yjjQ-bglJ* operon by LeuO may yield sufficient BglJ protein for activation of *P*_*leuO*_ we compared *P*_*leuO*_ *mVenus* expression in (*yjjQ-bglJ*)^+^ strain U95 with expression in the isogenic Δ(*yjjQ-bglJ*) strain U69 (Figure 3). The data revealed no difference between wild-type *yjjQ-bglJ*^+^ strain U95 and Δ(*yjjQ-bglJ*) strain U69 suggesting that activation of *yjjQ-bglJ* by LeuO is either too low to provide sufficient levels of BglJ for activation of *P*_*leuO*_ *mVenus* or that LeuO interferes with activation by BglJ-RcsB. Second, we analyzed whether the presence of native *leuO* may affect activation of the *leuO* promoter by BglJ-RcsB. For this analysis the *leuO* gene was retained at its native locus and the fluorescence reporter gene *mVenus* was inserted downstream of *leuO* (as a transcriptional fusion) resulting in allele *P*_*leuO*_ *leuO::mVenus* in strain U97. Transformants of this strain with *bglJ* carrying plasmid pKETS26 (*P*_*UV*5_ *bglJ*, pSC-*ori*), were grown with IPTG concentrations ranging from 10 μM to 200 μM and *P*_*leuO*_ *leuO::mVenus* expression was determined by flow cytometry. Comparison of the data obtained of *P*_*leuO*_ *leuO::mVenus* with the data obtained for *P*_*leuO*_ *mVenus* (Δ*leuO*) revealed no significant difference (Figures 2B,D). These data indicate that induction of the native *leuO* gene by BglJ does not provide sufficient LeuO to antagonize BglJ-RcsB-mediated activation of *leuO*.

**Figure 7:**
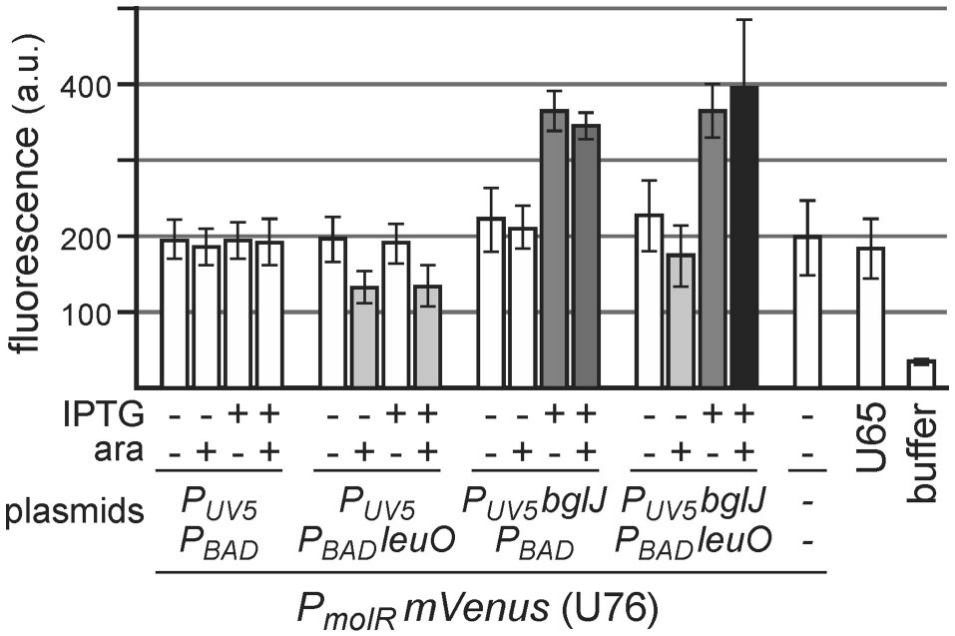
**Activation of the *molR* promoter (*P*_*molR*_) by BglJ-RcsB is not affected by LeuO**. For determining activation of *P*_*molR*_ by BglJ-RcsB strain U76 was used that carries a replacement of the *molR* coding region by *mVenus*. Transformants of U76 with plasmids carrying *P*_*UV*5_ *bglJ* (pKETS26) and *P*_*tac*_ *leuO* (pKES303) as well as control plasmids (pKETS24 and pBAD30) were grown in tryptone medium for 5 h. For induction (+) IPTG (100 μM) and arabinose (50 μM) were added. When harvested, the cultures had an OD_600_ of approximately 1, while induction of *leuO* resulted in slower growth to OD_600_ of approximately 0.6. Yellow fluorescence of three biological replicates was determined and expression levels are given in arbitrary units (a. u.).

Furthermore, we analyzed whether LeuO inhibits BglJ-RcsB-mediated activation of *leuO* transcription indirectly by downregulating BglJ-RcsB activity rather than by inhibiting activation of the *leuO P2* promoter by BglJ-RcsB. To this end, activation of another BglJ-RcsB-activated promoter, the *molR* promoter (Salscheider et al., 2014), was analyzed in absence and presence of LeuO. BglJ was provided by *P*_*UV*5_ *bglJ* plasmid pKETS26, and LeuO was provided by *P*_*BAD*_ *leuO* plasmid pKES303. As control, transformants with the empty vectors were analyzed in parallel. Activity of the *molR* promoter was determined using a *P*_*molR*_ *mVenus* reporter fusion. The expression analyses demonstrate that LeuO neither does affect activation of *P*_*molR*_ by BglJ-RcsB nor does LeuO-mediated activation of the native *yjjQ-bglJ* operon present in strain U76 lead indirectly to activation of *P*_*molR*_ (Figure 7). We note that induction of the *P*_*BAD*_ *leuO* with 50 μM arabinose resulted in slower growth to OD_600_ = 0.6 after 5 h as compared to OD_600_ = 1 which may explain the 1.5-fold reduce in basal expression of *P*_*molR*_ *mVENUS* in transformants of *P*_*BAD*_ *leuO* plasmid pKES303 and control plasmid *P*_*UV*5_ pKETS24 (Figure 7).

## Discussion

In *E. coli* transcription of *leuO* is directed by at least two promoters, *P1* and *P2*, which are repressed by H-NS and StpA. The *P2* promoter requires activation by BglJ-RcsB, while LeuO inhibits activation of *P2* by BglJ-RcsB. In addition, LeuO represses the *leuO* promoters in *hns stpA* mutants. Thus, *leuO* is antagonistically regulated by BglJ-RcsB and LeuO. The characterization of *leuO* transcription using a *leuO* promoter-*mVenus* reporter fusion revealed that the antagonistic regulation of *leuO* transcription by LeuO and by BglJ-RcsB correlates to the relative cellular amounts of these regulators. The experimental data are in agreement with a theoretical model according to which LeuO and BglJ-RcsB regulate transcription independently. Further, data indicate that double-positive feedback regulation of *leuO* and *bglJ* is of minor relevance, at least at the laboratory steady state conditions tested, since deletion of *leuO* and *bglJ*, respectively, had no significant effect on the regulation of the *leuO* promoter reporter fusion by LeuO and BglJ-RcsB.

Activation of the *leuO P2* promoter by the BglJ-RcsB heterodimer does not occur under standard lab conditions due to H-NS-mediated repression of the *yjjQ-bglJ* operon (Stratmann et al., 2008, 2012). To address the antagonistic regulation of *leuO* transcription by BglJ-RcsB and LeuO, we tested low to medium copy plasmids for gradual induction of *bglJ* under control of the *P*_*UV*5_ and *P*_*BAD*_ promoter, respectively. The data show that rather low amounts of BglJ are sufficient for full activation of the *leuO P2* promoter (Figures 2, 4, 5). Gradual activation of *leuO* by BglJ-RcsB was observed only upon gradual induction of *bglJ* provided by the low-copy *P*_*UV*5_ *bglJ* plasmid, while *bglJ* expression levels directed by the *P*_*BAD*_ *bglJ* plasmid turned out to be too high even when induced with just 2 μM arabinose, while induction with 100 μM arabinose caused growth defects. Likewise, we addressed autoregulation of *leuO* transcription by gradual induction of *leuO* carrying plasmids, which carry *leuO* under control of the *P*_*UV*5_, *P*_*tac*_, and *P*_*BAD*_ promoter, respectively. The data (Figure 3) indicate that positive autoregulation of *leuO* that was reported previously (Fang and Wu, 1998; Chen et al., 2003; Stratmann et al., 2012) is negligible at steady state growth conditions.

Further experiments, with simultaneous gradual induction of *bglJ* and *leuO* revealed that the activity of the *leuO* promoter correlates with the relative BglJ and LeuO concentrations (Figure 5). Interestingly, no switch-like response was observed. This might be plausible, because the distance of the LeuO DNA-binding sites to the BglJ-RcsB DNA-binding site is more than 100 bp (Stratmann et al., 2012), and LeuO and BglJ-RcsB presumably can bind simultaneously. Therefore, the LeuO-mediated inhibition of activation by BglJ-RcsB is putatively not caused by competition for binding, but by another mechanism, as for example inhibition of RNA polymerase binding to *leuO* promoter *P2* or inhibition of transcription initiation at *P2* by LeuO. Such a mechanism of repression is supported by *in vitro* DNA binding analyses, which revealed that LeuO inhibits open complex formation by RNA polymerase at sites mapping next to *leuO* promoter *P1* and reduces open complex formation by RNA polymerase at sites close to *P2* (Stratmann et al., 2012). A thermodynamic model based on Michaelis-Menten kinetics (Figure 6) supports the interpretation that antagonistic regulation by BglJ-RcsB and LeuO is mediated by independent mechanisms.

Previous data suggested that LeuO is controlled by interlocked double-positive and negative feedback control, because LeuO activates expression of the H-NS repressed *yjjQ-bglJ* operon (Stratmann et al., 2008). In the present study we analyzed whether activation of *bglJ* by LeuO may indirectly also turn on transcription of *P*_*leuO*_ *mVenus* (Figure 3) or *P*_*molR*_ *mVenus* as another BglJ-RcsB target (Figure 7), which was not the case indicating that activation of the native *yjjQ*-*bglJ* operon by LeuO does not yield sufficient BglJ. Likewise, expression analyses of an *mVenus* fusion downstream of the *leuO* coding region yielded the same results as the *P*_*leuO*_ *mVenus* reporter indicating that LeuO levels, when expressed from its native locus, remain too low to antagonize BglJ-RcsB. Taken together, double-positive feedback regulation of the *leuO* and *yjjQ-bglJ* loci is not relevant, at least at laboratory conditions, since the presence of the native *leuO* gene had no effect on BglJ-RcsB mediated activation of *leuO* that was triggered by plasmidic *bglJ*. Likewise the presence of native *bglJ* had no influence. Thus, the data suggest that repression of *leuO* by H-NS and StpA and of *yjjQ-bglJ* by H-NS dominates regulation of these loci and keeps them in the OFF state.

## Materials and methods

### Strains, media, and plasmids

Bacterial cultures of *E. coli* K-12 were grown in LB (10 g/l Bacto Tryptone, 5 g/l Bacto Yeast Extract, 5 g/l NaCl) or tryptone (10 g/l Bacto Tryptone, 5 g/l NaCl) media. Antibiotics were added with concentrations of 50 μg/ml ampicillin, 15 μg/ml chloramphenicol, and 25 μg/ml kanamycin. Strains, listed in Table 1, were constructed by transduction using phage T4*GT7*, by Red-Gam mediated gene deletion or gene replacement, and by homologous recombination, as described (Wilson et al., 1979; Hamilton et al., 1989; Datsenko and Wanner, 2000). Plasmids and their construction are listed in Table 2 and oligonucleotides are listed in Table 3. Standard molecular techniques, such as cloning, PCR, culture growth and induction of plasmid-provided genes, were performed according to standard protocols (Ausubel et al., 2005).

**Table 2:** **Plasmids**.

**Plasmid**	**Features^a^**	**Reference, Construction**
pBAD30	araC P_BAD_ MCS ori-p15A amp	Guzman et al., 1995
pKD3	FRT cm FRT oriRγ amp	Datsenko and Wanner, 2000
pKD4	FRT kan FRT oriRγ amp	Datsenko and Wanner, 2000
pKD46	P_BAD_ λ-Red-recombinase amp (rep^ts^ ori-pSC)	Datsenko and Wanner, 2000
pCP20	cI_857_ λ-P_*R*_ flp-recombinase cm amp (rep^ts^ ori-pSC)	Cherepanov and Wackernagel, 1995
pVS133	mVenus (*yfp* variant) in pTrc99a	V. Sourjik laboratory, Germany, and (Amann et al., 1988)
pKESK10	lacI PUV5 bglG ori-pSC cm	Dole et al., 2002
pKESK22	lacI^q^ P_tac_ MCS in ori-p15A kan	Stratmann et al., 2008
pKETS1	lacI^q^ P_tac_ bglJ in pKESK22 (ori-p15A kan)	Venkatesh et al., 2010
pKETS5	lacI^q^ P_tac_ leuO in pKESK22 (ori-p15A kan)	Stratmann et al., 2012
pKETS27	chi-site polB' ΔaraDABC yabI chi-site tetR (rep^ts^ ori-pSC)	fragments flanking *araC-BAD* were amplified by PCR with T646/T647 and T648/T649, and cloned into a tetR rep^ts^ ori-pSC vector, chi-sites were included to enhance homologs recombination
pKETS28	chi-site cynX Δ lacAYZI mhpR chi-site tetR (rep^ts^ ori-pSC)	fragments flanking *lacI-lacZYA* were amplified by PCR with T650/T651 and T652/T653, and cloned into a tetR rep^ts^ ori-pSC vector, chi-sites were included to enhance homologs recombination
pKES285	pKD3 with MCS (BamHI SpeI EcoRI SalI)	pKD3 (NdeI) × annealed oligos T540/T541
pKES287	pKD4 with MCS (BamHI SpeI EcoRI SalI)	pKD4 (NdeI) × annealed oligos T540/T541
pKES292	mVenus (with enhanced RBS^b^) in pKD3	mVenus fragment amplified by PCR with T146/T368 of pVS133, digested with BamHI, EcoRI cloned into BamHI, EcoRI-digested vector plasmid pKES285
pKES293	mVenus (with enhanced RBS) in pKD4	mVenus fragment cloned as pKES292, but into vector plasmid pKES287
pKES302	araC P_BAD_ bglJ in pBAD30 (ori-p15A amp)	*bglJ* fragment of pKETS1 (EcoRI, XbaI) cloned into pBAD30 (EcoRI, XbaI)
pKES303	araC P_BAD_ leuO in pBAD30 (ori-p15A amp)	*leuO* fragment generated by PCR with primers S326/T558, EcoRI and XbaI digested, and cloned into pBAD30 (EcoRI, XbaI)
pKETS25	lacI P_UV5_ leuO ori-pSC cm	*leuO* fragment generated by PCR with primers T644/T645 of pKETS5, digested with EcoRI and BamHI, and cloned into EcoRI, BamHI digested pKESK10
pKETS26	lacI P_UV5_ bglJ ori-pSC cm	cloning of *bglJ* fragment of pKETS1 (BamHI, EcoRI) into BamHI, EcoRI digested pKESK10
pKEHB27	lacI^q^ P_tac_ leuO ori-pSC cm	replacement of *lacI* P_UV5_ in pKETS25 by *lacI^q^* P_tac_ fragment of pKESK22
pKEHB28	lacI^q^ P_tac_ bglJ ori-pSCori cm	replacement of *lacI* P_UV5_ in pKETS26 by *lacI^q^* P_tac_ fragment of pKESK22
pKEHB29	araC P _ara_ mVenus in pBAD30 (ori-p15A amp)	mVenus fragment of pVS133 cloned in pBAD30 (EcoRI, XbaI)

a The following abbreviations and genetic designations are used: FRT, Flp recombinase target site; MCS, multiple cloning site; genes coding for antibiotic resistance are designated as amp, ampicillin resistance, cm, chloramphenicol resistance, kan, kanamycin resistance. Origins of replications include ori-pSC (derived of low-copy plasmid pSC101), ori-p15A (derived of low to medium copy plasmid p15A), and Pir-dependent oriRy.

b m Venus was fused to the enhanced RBS (ribosomal binding site) that is derived of phage T7, gene 10 (Olins and Rangwala, 1989).

**Table 3:** **Oligonucleotides**.

**Oligo**	**Sequence^a^**	**Purpose**
S326	aagaattcggatccGTGTGACAGTGGAGTTAAGTATGCCAG	*leuO* fragment
S911	TTTGTTCATGCCGGATGCGGCTAATGTAGATCGCTGAACTgtgtaggctggagctgcttcg	construction of Δ(*lacI-lacZYA*)
S937	ATGATAGCGCCCGGAAGAGAGTCAATTCAGGGTGGTGAATcatatgaatatcctccttagttcctattcc	construction of Δ(*lacI-lacZYA*)
T146	ctgaagcttgctagctcgaggaattcaataattttgtttaactttaagaaggagatatacatATGAGCAAGGGCGAGGAGCTG	mVenus amplification from pVS133
T368	cgatggatccaattgtctagaTTACTTGTACAGCTCGTCCATGCC	mVenus amplification from pVS133
T540	TAGGATCCATACTAGTAAGAATTCGTGTCGAC	MCS
T541	TAGTCGACACGAATTCTTACTAGTATGGATCC	MCS
T547	CAGTGGATGGAAGAGCAATTAGTCTCAATTTGCAAACGCTAAttcaataattttgtttaactttaagaaggagatatacat	mVenus integration at *leuO*
T548	TAAACCAGACATTCATGTCTGACCTATTCTGCAATCAGgtgtaggctggagctgcttcg	mVenus integration at *leuO*
T558	agtgtctagaTGACCTATTCTGCAATCAGTTAGCG	*leuO* fragment
T585	TTTATATGCATGATAAATCATATTCTTCAGGATTATTTCTCTGCATTCCAttcaataattttgtttaactttaagaaggagatatacat	*leuO* replacement by mVenus
T644	gaccgaattcGTGTGACAGTGGAGTTAAGTATGCCAG	*leuO* fragment
T645	aggtggatccTGACCTATTCTGCAATCAGTTAGCG	*leuO* fragment
T646	gaccctgcagGCTGGTGGGACCAAATGCCGCCACCGA	for *araC-BAD* deletion
T647	gaccgaattcTAATGACTGTATAAAACCACAGCCAATC	for *araC-BAD* deletion
T648	gaccgaattcTAATTGGTAACGAATCAGACAATTGACG	for *araC-BAD* deletion
T649	gacctctagaGCTGGTGGACAAGACTATCTCCTAAACCCCAACC	for *araC-BAD* deletion
T650	gaccctgcagGCTGGTGGGTGCTGATTGGTCTTAATATGCGACC	for *lacI-ZYA* deletion
T651	gaccgaattcAGTTCAGCGATCTACATTAGCCGCA	for *lacI-ZYA* deletion
T652	gaccgaattcATTCACCACCCTGAATTGACTCTCTTC	for *lacI-ZYA* deletion
T653	gacctctagaGCTGGTGGTAACAGCAGGCTGGATGTCAGGG	for *lacI-ZYA* deletion
T946	CGCATAAATACTGGTAGCATCTGCATTCAACTGGATAAAATTACAGGGATGCAGAaataattttgtttaactttaagaaggagatatacatat	mVenus integration at *molR*
T947	GTTGGGCGTTATCCGCCAGCCACGGTAATTCCTTGTCCATGCTCTTTCCgtgtaggctggagctgcttcg	mVenus integration at *molR*

a Sequences homologous to the indicated target loci are printed in capital letters, sequences in lower case that map at the 3′ ends serve for annealing to the pKD3 and pKD4 derived template plasmids pKES292 and pKES293 to generate PCR fragments for Red-Gam mediated integration. In addition, 5′ extensions of oligonucleotides are shown in lower case letter, restriction endonuclease sites are underlined, and chi-sites are underlined and shown in upper case letters.

### Flow cytometry and fluorescence assay

For expression analyses by flow cytometry cultures of transformants were inoculated from fresh overnight cultures to an OD_600_ of 0.05 and grown for 5 h at 37°C in 10 ml tryptone medium containing antibiotics for selection of the plasmids. The cultures were diluted to OD_600_ of 0.1 and kept on ice prior to analysis by flow cytometry. Flow cytometry was performed on a BD FACScalibur flow cytometer using CellQuest software (BD Biosciences, Franklin Lakes, NJ, USA). For each sample, 50,000 events were measured at a rate between 500 and 1000 events per second. The experiments were repeated at least twice and representative sets of data are shown.

Fluorescence directed by the *P*_*molR*_ *mVenus* fusion was determined by Fluorescence spectroscopy using a CLARIOstar plate reader (BMG LABTECH, Germany). Briefly, cultures were grown as for flow cytometry and the fluorescence of cells equivalent to 1.5 OD_600_ was measured using yellow fluorescent proteins specific excitation (495–515 nm) and detection (540–620 nm) channels. The average obtained of three biological replicates was calculated and the standard deviation is less than 25%.

### Theoretical model

To describe the transcription rate directed by PleuO in dependence of the concentration of BglJ and LeuO, a thermodynamic model based on Michaelis-Menten kinetics was used. In this model it was assumed that BglJ and LeuO regulate PleuO independently of each other. The binding probabilities were defined as B/(Bo+B) and L/(Lo+L), where B represents the concentration of BglJ in the cell, B0 the BglJ concentration at which the promoter is half occupied, L represents the concentration of LeuO and L0 the LeuO concentration at which the promoter is half occupied. Since LeuO acts as a repressor and BglJ as an activator of the leuO promoter four different states with a different expression rate were described. The basal expression level directed by PleuO in absence of BglJ and LeuO was defined as η0. In presence of LeuO and absence of BglJ, expression remains at a basal level defined as η0. However, in presence of BglJ but absence of LeuO, the expression level is higher which is defined as η1. When BglJ and LeuO are bound at the same time, the expression rate is defined as η0, because high levels of LeuO inhibit activation by BglJ, when BglJ is provided by the low-copy *P*_*UV*5_ *bglJ* plasmid. Taking these four different states into account the expression rate of leuO in dependence of LeuO and BglJ concentration was described as

$$\eta_{B_{0},L_{0}}\left(B,L\right)=\frac{\eta_{0}+\eta_{0}\frac{L}{L_{0}}+\eta_{1}\frac{B}{B_{0}}+\eta_{0}\frac{L}{L_{0}}\frac{\text{B}}{{\text{B}}_{\text{0}}}}{\left(\;1\;+\;\frac{B}{B_{0}}\right)\left(\;1\;+\;\frac{L}{L_{0}}\right)}$$

The function was fitted to the median expression values determined by flow cytometry (*P*_*UV*5_ *bglJ*, and *P*_*BAD*_ *leuO*, Figure 5) using non-linear regression according to (Fox and Weisberg, 2011), which yielded a high fitting significance (*P-value* < 0.001).

## Author contributions

HB contributed to the design of the work, acquired the data, and together with KS interpreted the data and drafted the work. KS conceived the project, contributed to the design of the work, and drafted the work.

## Funding

Funding was obtained by the Deutsche Forschungsgemeinschaft through grant SCHN 371/10-2.

### Conflict of interest statement

The authors declare that the research was conducted in the absence of any commercial or financial relationships that could be construed as a potential conflict of interest.
